# Decreased and Improved Movement Abilities in a Case of Myotonic Dystrophy Type 1: Examining Longitudinal Characteristics Based on Repeated Evaluations

**DOI:** 10.7759/cureus.60818

**Published:** 2024-05-21

**Authors:** Kiyoshige Ishibashi, Daisuke Ishii, Satoshi Yamamoto, Yusuke Ono, Kenichi Yoshikawa, Tomoyuki Matsuda, Yasutsugu Asakawa, Yutaka Kohno

**Affiliations:** 1 Department of Physical Therapy, Ibaraki Prefectural University of Health Sciences Hospital, Ami-machi, JPN; 2 Department of Occupational Therapy, Ibaraki Prefectural University of Health Sciences Hospital, Ami-machi, JPN; 3 Department of Cognitive Behavioral Physiology, Graduate School of Medicine, Chiba University, Chiba, JPN; 4 Center for Medical Sciences, Ibaraki Prefectural University of Health Sciences, Ami-machi, JPN

**Keywords:** rehabilitation, neuromuscular disease, muscle weakness, functional compensation, disuse syndrome

## Abstract

Several large longitudinal studies on myotonic dystrophy type 1 (DM1) patients have revealed that proximal muscles show more gradual muscle weakness than distal muscles and that the progression of muscle weakness might differ between the sexes. However, these longitudinal studies were based on two follow-up time points. The present report aimed to verify the longitudinal characteristics of muscle strength and various movement abilities in a case of DM1 by examining the results of 44 repeated evaluations for approximately two years. A 40-year-old male patient with DM1 could walk independently without any aid. We recorded the longitudinal changes in his muscle strength and movement ability during outpatient rehabilitation. During follow-up, he had a fall and was diagnosed with a right ankle sprain. To evaluate the effects of the fall, we examined his recorded data. He had a significant decrease in right knee extensor muscle strength after the fall, suggesting muscle weakness due to disuse syndrome. Although his right knee extensor muscle strength and walking speed decreased, the timed up-and-go test score was improved, and walking endurance in the 2-minute walk test was maintained. In the present case, there were some motor tasks in which the movement ability was maintained or improved, likely due to the use of compensation by residual function, even when muscle weakness was present. Regular and repeated evaluations of patients with DM1 lead to reveal longitudinal characteristics of their dysfunction and movement ability.

## Introduction

Myotonic dystrophy type 1 (DM1), the most prevalent inherited neuromuscular disease in adults [1,2], is a progressive, multifaceted disorder affecting multiple organs, including the muscle, respiratory, cardiac, endocrine, ocular, and central nervous systems [3]. Therefore, the clinical presentations of DM1 are diverse and complex, and its clinical management is often challenging [4].

Several large longitudinal studies have explored the natural history of physical function in patients with DM1 [5-10]. Particularly, regarding the major symptom of muscle weakness, these studies have revealed that proximal muscles show more gradual muscle weakness than distal muscles [6,7,9,10] and that the progression of muscle weakness might differ between sexes [7,9,10]. These longitudinal studies [5-10] are fragmented, based on two follow-up time points. Furthermore, a cross-sectional study on the relationship between lower extremity muscle strength and movement ability in patients with DM1 has reported that knee extensors and ankle dorsiflexors showed a strong relationship with performance scores, such as those in the 10-minute walk test (10 mWT) and timed up and go (TUG) test [11].

In the present report, we conducted repeated evaluations of muscle strength and movement abilities over approximately two years of outpatient rehabilitation. As a result, we encountered a patient with DM1 whose right knee extensor muscle strength decreased after a fall. This patient exhibited decreased gait speed in 10 mWT after a fall. Contrarily, improvement was observed in the TUG test. Regular and repeated evaluations may uncover longitudinal characteristics of dysfunction and movement ability in patients with DM1 that have not been detected so far, and help clarify rehabilitation strategies, such as interventions to prevent the progression of dysfunction and/or to maintain movement ability.

This study aimed to verify the longitudinal characteristics of the muscle strength and various movement abilities of a patient with DM1 by examining the results of repeated evaluations for approximately two years.

## Case presentation

A 40-year-old male who was aware of the muscle weakness in his lower limbs at the age of 36 years was confirmed to have DM type 1 by genetic testing. He was able to independently perform all activities of daily living (ADLs) and could walk independently without the use of aids or braces. He worked in a clerical position and commuted to work in a car that he drove himself. To evaluate the longitudinal changes in his muscle strength and movement ability, he underwent outpatient rehabilitation at our hospital twice a month, and his muscle strength (bilateral knee extensor muscle strength and grip strength) and movement ability (10 mWT, TUG test, 2-minute walk test, and two-step distance from a stationary standing position) were recorded.

For the evaluation of muscle strength, a hand-held dynamometer (Salt Lake City, UT: Hoggan Scientific, LLC.; microFET2) was used to measure knee extensor muscle strength. The patient was seated in a backless chair and performed isometric knee extensions with maximal effort from a position of 90º of hip and knee flexion. The hand-held dynamometer was firmly fixed by the evaluator just above his ankle joint and perpendicular to the lower leg axis. Two trials were performed for each of the right-left orders. Grip strength was evaluated using a grip dynamometer (Niigata, Japan: Takei Scientific Instruments Co., Ltd.; GRIP-D), and he performed two trials each in the right-left order with maximal effort. The maximum value of each data obtained was recorded as the representative value of the day.

Among the tests performed to evaluate movement ability, the 10 mWT was performed using a 16-m walking path with 3-m acceleration and deceleration sections at both ends. He performed two trials of walking at a comfortable speed without aids or braces. For the TUG test, we measured the time required for the patient to get up from a chair without armrests, go around a cone placed 3 m away, and sit down in the chair. He was asked to walk at a comfortable speed without aids or braces, and two trials were performed. The 2-minute walk test (2 mWT) was performed in the corridor and the distance he walked in 2 minutes was measured. He was asked to walk as long a distance as possible without aids or braces, taking care to avoid falling. To minimize fatigue, only one 2 mWT was performed. The 2-step distance from a stationary standing position (2SD) was performed in parallel bars and the distance taken in two steps from a stationary standing position was measured. He was required to step as far as possible in two trials without any aids or braces, taking care to avoid falling. For each of the three evaluations, except the 2 mWT, the better value of each data obtained was recorded as the representative value for the day.

During his outpatient rehabilitation follow-up in our hospital, he fell at an aquarium that he visited on a family vacation. During his fall, the aquarium was very crowded, and the dark inside made it difficult for him to see his feet. After the fall, his right ankle had residual loading pain; therefore, he visited a local orthopedic surgeon and was diagnosed with a sprain of the right ankle. The patient was required to use a crutch for two weeks to support loading on his right leg and rest of the right ankle for three weeks. Approximately a month after the fall, the pain in his right ankle was gone, but he sometimes noticed lower limb muscle weakness. He continued our outpatient rehabilitation but developed pneumonia in late November of the same year and was hospitalized at another hospital. After recovering from pneumonia, he missed outpatient rehabilitation because of work and New Year’s holidays. Subsequently, the outpatient rehabilitation in our hospital was stopped because of the coronavirus disease pandemic.

To evaluate the effects of the fall and subsequent rest on his muscle strength and movement ability and to verify these longitudinal characteristics over approximately two years, we examined the longitudinal changes in muscle strength and movement ability from the beginning of the outpatient rehabilitation to the period after the fall. Each of the recorded data was divided into the following four periods based on the date of the fall and each period was compared: 1.5 years to one year before the fall (Period A), one year to six months before the fall (Period B), six months before the fall to the date of the fall (Period C), and after the fall (Period D). Additionally, to clarify the relationship between knee extensor muscle strength and body weight, total knee extensor strength was calculated using the following formula: total knee extensor strength_(N/kg)_ = (right knee extensor muscle strength_(N)_ + left knee extensor muscle strength_(N)_) / body weight_(kg)_

Given that each recorded data showed an autocorrelation, the Kruskal-Wallis and Bonferroni correction tests were used for multiple comparisons. SPSS software (Armonk, NY: IBM Corp.) was used for the statistical analyses and the significance level was set at p<0.05.

**Figure 1:**
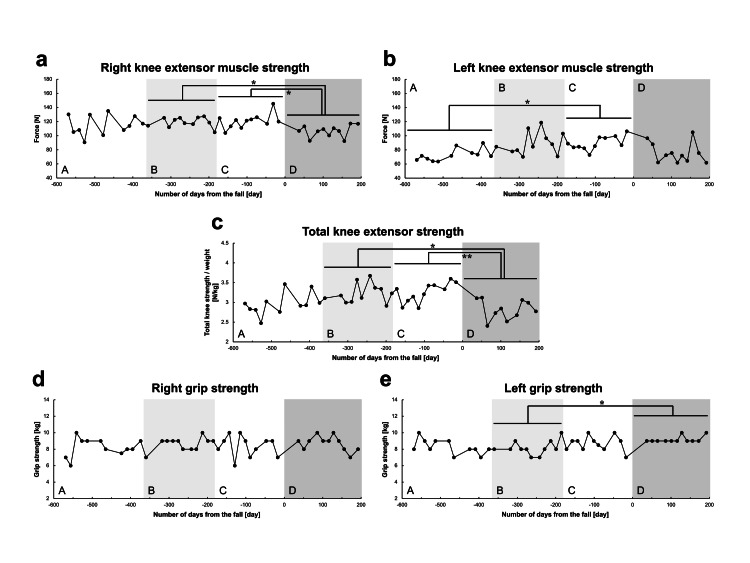
Longitudinal changes in muscle strength. (a) Right knee extensor muscle strength. (b) Left knee extensor muscle strength. (c) Total knee extensor strength. (d) Right grip strength. (e) Left grip strength. The horizontal axis of each graph shows the number of days from the fall (i.e., the date of the fall is 0, the period before the fall is shown as negative days, and the period after the fall is shown as positive days). Based on the date of the fall, Periods A, B, C, and D represent the period 1.5 years to one year before the fall, one year to six months before the fall, six months before the fall to the date of the fall, and after the fall, respectively. *P-value <0.05. **P-value <0.01.

Figure 1 shows the longitudinal changes in muscle strength. The right knee extensor muscle strength was significantly decreased in Period D (after the fall) as compared with those in Periods B (one year before the fall, p=0.037) and C (six months before the fall, p=0.033) (Figure 1a). The left knee extensor muscle strength increased significantly in Period C as compared with that in Period A (1.5 years before the fall, p=0.020), although no significant change was observed in Period D after the fall (Figure 1b). The total knee extensor strength was significantly decreased in Period D after the fall as compared with those in Periods B (p=0.016) and C (p=0.009) (Figure 1c). No significant change was observed in the right grip strength (Figure 1d), although there was a significant increase in the left grip strength in Period D as compared with Period B (p=0.020) (Figure 1e).

**Figure 2:**
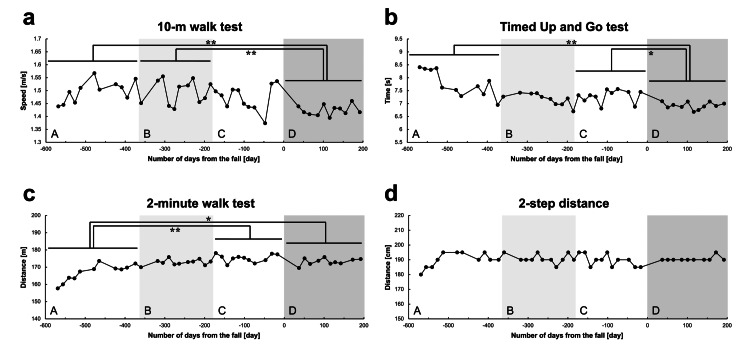
Longitudinal changes in movement ability. (a) 10-meter walk test. (b) Timed up and go test. (c) 2-minute walk test. (d) 2-step distance. The horizontal axis of each graph shows the number of days from the fall (i.e., the date of the fall is 0, the period before the fall is shown as negative days, and the period after the fall is shown as positive days). Based on the date of the fall, Periods A, B, C, and D represent the period 1.5 years to one year before the fall, one year to six months before the fall, six months before the fall to the date of the fall, and after the fall, respectively. *P-value <0.05. **P-value <0.01.

Figure 2 shows the longitudinal changes in the movement ability. The 10 mWT showed a significant decrease in Period D after the fall as compared with those of Periods A (p=0.002) and B (p=0.002) (Figure 2a). Contrarily, the TUG test score showed a significant improvement in Period D after the fall as compared with Periods A (p<0.001) and C (p=0.032) (Figure 2b). The 2 mWT showed significant improvement in Periods C (p<0.001) and D (p=0.017) as compared with Period A (Figure 2c), but the 2SD showed no significant change in each period (Figure 2d).

The authors explained the purpose of the present study orally to the patient and obtained his written consent.

## Discussion

In the present report, we verified the longitudinal characteristics of muscle strength and various movement abilities in a patient with DM1 by examining the results of repeated evaluations for approximately two years. As a result, a significant decrease was observed in the right knee extensor muscle strength in Period D after the fall, suggesting muscle weakness due to disuse syndrome. Moreover, in Period D after the fall, 10 mWT showed a decrease in gait speed, whereas the TUG test score showed an improvement, and there were discrepancies between the decrease in right knee extensor muscle strength and the decrease in some movement abilities. These results suggest that there are some motor tasks whose movement ability can be maintained or improved in patients with DM1, even when they have weak muscle strength. Regular and repeated evaluations, as in the present case, uncovered the longitudinal characteristics of dysfunction and movement ability in DM1 that have not been detected so far.

Although there have been several reports of longitudinal changes in physical function over time in patients with DM1 with two follow-up time points, to the best of our knowledge, reports of longitudinal changes with repeated evaluations as in the present report are lacking [5-10]. Our case showed a decrease in the right knee extensor muscle strength in Period D, which is the post-fall period. The cause of this muscle weakness is unclear, although it is probably related to disuse syndrome due to the rest required for the treatment of the right ankle sprain. In cases of muscular diseases, deconditioning and disuse syndrome resulting from decreased physical activity are thought to provoke muscle weakness beyond the disease progression [12,13], and the necessity of its prevention has been advocated [14]. Nevertheless, to the best of our knowledge, there have been no reports based on case data of muscle weakness resulting from disuse syndrome in patients with DM1. The present report is valuable as our case data suggest muscle weakness resulting from disuse syndrome in patients with DM1 and support the importance of preventing disuse syndrome in patients with DM1 as an intervention to prevent the progression of dysfunction.

Additionally, the left knee extensor muscle strength (Period A vs. Period C) and left grip strength (Period B vs. Period D) were improved in our patient. As there were no events affecting his physical activity in periods A, B, and C, the factors contributing to these improvements are unknown. However, regarding the improvement in left grip strength, the use of a crutch with his left hand during the right leg partial loading period may have contributed to the muscle strength improvement. Although a previous study has reported no effect of muscle strength training on patients with DM1 [15], others have reported increased muscle strength [16-18]. Our case is considered to be in the early stage of muscle weakness because the patient was able to walk independently without the use of aids or braces and was able to independently perform all ADLs. Further elucidation of the detailed longitudinal characteristics of patients with DM1, such as at what stage of the DM1 is more responsive to muscle strength training, would be expected.

In our case, the patient’s walking speed decreased in Period D, when the right knee extensor muscle and total knee extensor strengths were decreased, whereas the TUG test score showed an improvement. The walking distance in the 2 mWT and the distance in the 2SD also showed no decrease in Period D after the fall. In a report examining the relationship between lower extremity muscle strength and mobility tests among patients with DM1, the analysis using regression models showed that the maximal isometric muscle strength of the knee extensor muscle was a significant explanatory variable for 10 mWT of comfortable speed and TUG. The results of the present case are inconsistent with the findings of the previous report, probably because the previous report was a cross-sectional study and did not reflect the longitudinal changes. When disuse syndrome occurs in patients with DM1, muscle weakness and resulting decrease in walking speed may arise earlier than the decrease in other movement tasks. In other words, the series of sequential movement tasks, such as TUG and the walking distance of the 2 mWT, may be less likely to decrease the capacity. In fact, longitudinal reports involving patients with DM1 have implied the different paces of muscle weakness and decreased movement ability, and it has been speculated that one of the factors contributing to this difference is compensation by residual function [10]. Among patients with DM1 with slow progression of muscle weakness, maintaining or improving movement ability may be possible using compensation by residual function as an intervention to maintain the ability, even when the muscle strength decreases.

As a limitation of this report, follow-up after Period D was not performed. Additionally, muscle strength other than the knee extensors and grip strength have not been evaluated. In particular, in patients with DM1, the relationship between ankle dorsiflexor muscle strength and movement ability as well as knee extensor muscle strength has been reported in cross-sectional studies [11,19]. The accumulation of detailed longitudinal data based on repeated evaluations of multiple lower extremity muscle strength and movement abilities in a large number of patients is expected in the future.

## Conclusions

In conclusion, in the present case, some motor tasks showed maintained or improved movement ability, likely through compensation by residual function, even when muscle weakness was present. Regular and repeated evaluations to capture detailed longitudinal changes in muscle strength and movement ability enable us to characterize the decline in ability in patients with DM1 and help clarify rehabilitation strategies, such as interventions to prevent the progression of dysfunction and/or to maintain movement ability.
